# Comparing Volume Loss in Neuroanatomical Regions of Emotion versus Regions of Cognition in Healthy Aging

**DOI:** 10.1371/journal.pone.0158187

**Published:** 2016-08-23

**Authors:** Peter S. Pressman, Yuliana Noniyeva, Nick Bott, Shubir Dutt, Virginia Sturm, Bruce L. Miller, Joel H. Kramer

**Affiliations:** Memory and Aging Center, Department of Neurology, University of California, 675 Nelson Rising Lane, Suite 190, San Francisco, California, 94158, United States of America; Yale University, UNITED STATES

## Abstract

Many emotional functions are relatively preserved in aging despite declines in several cognitive domains and physical health. High levels of happiness exist even among centenarians. To address the hypothesis of whether preservation of emotional function in healthy aging may relate to different rates of age-related volume loss across brain structures, we performed two volumetric analyses on structural magnetic resonance neuroimaging of a group of healthy aging research participants using Freesurfer version 5.1. Volumes selected as supporting cognition included bilateral midfrontal and lateral frontal gyri, lateral parietal and temporal cortex, and medial temporal lobes. Volumes supporting emotion included bilateral amygdala, rostral anterior cingulate, insula, orbitofrontal cortex, and nucleus accumbens. A cross-sectional analysis was performed using structural MRI scans from 258 subjects. We found no difference in proportional change between groups. A longitudinal mixed effects model was used to compare regional changes over time in a subset of 84 subjects. Again, there was no difference in proportional change over time. While our results suggest that aging does not collectively target cognitive brain regions more than emotional regions, subgroup analysis suggests relative preservation of the anterior cingulate cortex, with greater volume loss in the nucleus accumbens. Implications of these relative rates of age-related volume loss in healthy aging are discussed and merit further research.

## Introduction

The detrimental effects of aging on cognition are widely recognized [1]. While these changes sometimes reflect cerebrovascular disease or early stages of a neurodegenerative process such as Alzheimer’s disease, normal, healthy aging is also associated with declines in processing speed, attention and memory, even in the absence of any known vascular disease, obvious cortical insult, or proteinopathy such as misfolding of amyloid or tau [2–4].

In contrast to cognitive decline in older adults, many aspects of emotional functioning are maintained or even improved as part of healthy aging [5–8]. Despite shrinking social networks, decreasing cognitive agility, and declining physical health, older adults demonstrate an emotional well-being that is equal to or better than their younger counterparts. High levels of happiness exist even among centenarians [9], and older adults maintain the ability to generate and experience emotions and empathic responses to others [10, 11].

The retention and in some cases improvement of emotional functioning in the face of well-described decline in general health, social networks and cognitive function has been dubbed the emotion paradox of aging [6–8]. There have been several proposed explanations for this preservation of emotional function. Emotional preservation likely results from a combination of adaptation and circumstance. Older adults may adapt to the ravages of time with a learned ability to make use of attentional strategies. Laboratory-based studies have found that while older adults are more reactive to emotional stimuli and may recover more slowly from both positive and negative emotional events [10, 12], older adults are better able to regulate their emotions through situation selection and reappraisal tactics that enable them to view even negative situations in a positive light [5, 13].

More circumstantial explanations propose that the emotion paradox of aging is a biological consequence of the aging process. Within the brain, relative emotional preservation could be an incidental result of different cerebral regional rates of atrophy over time. For example, some studies have suggested that the anterior cingulate cortex is relatively preserved [14, 15], though such preservation is not universally described [16]. If true, the ACC could contribute to emotional health due to its involvement with selective attention and reappraisal [17]. Another circumstantial explanation for preserved emotional positivity in aging is that positive material may be less cognitively demanding than negative information [18]. As a result, systemic efficiency demands that energy be spent on less exhausting positive information, driving an active regulation of sensory input based on recognized cognitive resources. Such a theory suggests that structures involved with appraisal of information as positive or negative, such as the amygdala [19], or structures involved with emotion-related information processing and learning, such as the nucleus accumbens [20], could be especially important in maintaining a positive outlook during healthy aging. While some have suggested different rates of volume loss within these structures as a potential cause of emotional preservation with aging [6], referencing existing longitudinal volumetric analysis of healthy aging brains [14, 15], direct comparisons of volume loss in brain regions associated with cognition versus those of emotion have not been made.

To further investigate the neuroanatomical foundations of emotional preservation in aging, we compared brain regions historically associated with cognition to those associated with emotion. We applied a cross-sectional analysis to compare volumes of cognitive regions and emotional regions as a function of age, and also performed a longitudinal analysis of regional volumes as a function of time. We hypothesized that in healthy aging, the collective group of regions associated with emotional processing would lose volume more slowly than brain areas associated with cognitive functioning. A slower rate of volume loss in brain structures associated with emotion could potentially explain retained emotional acumen despite cognitive deterioration over time.

## Materials and Methods

### Participants

Research participants were enrolled from the Larry L. Hillblom Aging Study of the Memory and Aging Center of the University of California, San Francisco. All participants provided written and informed consent. The institutional review board of the University of California, San Francisco approved the study, and all investigations were conducted according to principles expressed in the Declaration of Helsinkini. Participants underwent a multidisciplinary assessment that included a neurological exam, neuropsychological testing, and structural MRI. Participants ranged from 60 to 100 years of age, with no significant subjective memory complaints, no functional impairment, a Clinical Dementia Rating (CDR) of 0, no diagnosis of mild cognitive impairment, and a Mini-Mental State Exam (MMSE) score of ≥ 26. Neuroimaging was collected on subjects from 2008 until 2013.

### Cross-sectional Study

258 individuals were included in the cross-sectional portion of our study. A neurological examination, cognitive testing, and interview with a reliable study partner who knew the participant well was performed at each visit in order to confirm the participants ongoing eligibility for inclusion. Each of these 258 individuals had a single MRI scan included in the cross-sectional analysis.

### Longitudinal Study

To follow-up on the cross-sectional study, we evaluated a subset of 84 subjects who received two to three scans (mean = 2.2, SD = 0.4). As scans were originally performed for various subprojects of the Larry L. Hillblom Foundation study, follow-up time varied between individuals. The average amount of time between scans was 1.9 years (SD = 0.8 years, min 0.8 years, max 4.4 years). At the time of each MRI, these participants were assessed as previously described in order to determine that they were still cognitively healthy and eligible for inclusion. Of those who enrolled, one went on to develop mild cognitive impairment, and one developed another health problem that excluded them from the study. Two people elected not to return after receiving two scans each.

### Magnetic Resonance Imaging

Structural neuroimaging was performed at every visit on a Siemens TIM Trio 3 Tesla MRI scanner located at the Neuroimaging Center of the University California, San Francisco. For anatomical analysis a T1-weighted 3D MP-RAGE sequence was used with 1 mm slice thickness, field of view 256 x 256 mm, matrix– 230 x 256, repetition time 2300 ms, echotime 2.98 ms, and a flip angle of 9°.

### Image Processing

For all research scans in this study, volumetric analysis was performed by trained research assistants using Freesurfer version 5.1 (http://surfer.nmr.mgh.harvard.edu/). Freesurfer is a semi-automated parcellation program in which cortical and subcortical volumes can be computed. Skull stripping, Tailarach transforms, atlas registration, spherical surface maps and parcellations were initialized with common information from the within–subject template [21]. The cortex was segmented using the Fischl and Desikan-Killiany atlases into 10 separate regional volumes of interest [21, 22]. Manual data quality checks were performed by trained researchers for each MR image to confirm proper segmentation, with manual corrections as deemed necessary by the technician.

### Regional Selection

While recognizing evidence that the right hemisphere is more involved with emotional processing generally, the functionality between the left and right hemispheres remains complex and poorly understood [23, 24]. Consequently, regions associated with cognition or emotion were selected bilaterally. The following were included as regions predominantly influencing cognition: the middle frontal and lateral frontal gyri, the parietal cortex, and the medial and lateral gyri of the temporal lobes [25–29]. The following were included as regions predominantly influencing emotion: amygdala, rostral anterior cingulate cortex, insula, orbitofrontal cortex (medial and lateral orbitofrontal cortex) and nucleus accumbens [30–35]. When we could not reasonably decide whether a region should be considered cognitive or emotional in nature, we excluded that region from the analysis. For example, the inferior temporal gyrus was not included due to its prominent involvement in semantic dementia, which has pronounced cognitive and emotional changes [36].

**Fig 1:**
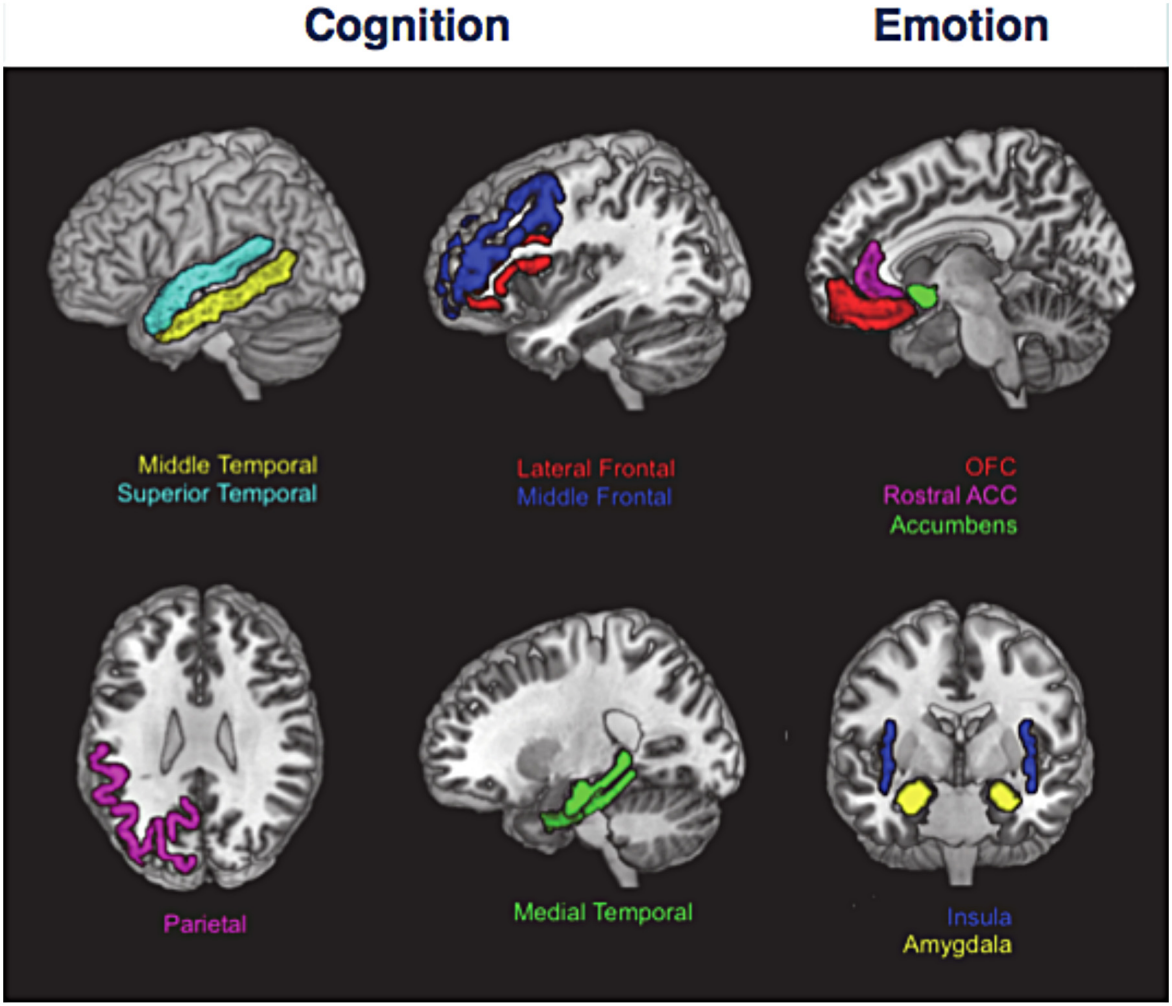
Regions included in cognitive and emotional collectives. For a listing of how these regions were developed, see Table 1.

In cases where these regions were not predefined from the atlas, regions were combined as needed. The orbitofrontal cortex measurement was obtained by summing the medial and lateral orbitofrontal cortex volumes. The middle frontal measurement summed the caudal middle frontal and rostral middle frontal volumes. The lateral frontal measurement summed the pars opercularis, pars orbitalis, and pars triangularis volumes. The parietal cortex summed the inferior parietal, superior parietal, precuneus, and supramarginal volumes. The lateral temporal region summed the superior and middle temporal gyrus volumes. The medial temporal region summed the hippocampal, entorhinal and parahippocampal volumes. (Fig 1, Table 1)

**Table 1:** List of regions used and source atlases.

Classification	Region of Interest	Contributing Regions	Source Atlas
emotion	Amygdala	.	Fischl
emotion	rostral anterior cingulate cortex	.	Desikan
emotion	Insula	.	Desikan
emotion	orbitofrontal cortex	medial orbitofrontal	Desikan
emotion	.	lateral orbitofrontal	Desikan
emotion	nucleus accumbens	.	Fischl
cognitive	middle frontal	caudal middle frontal	Desikan
cognitive	.	rostral middle frontal	Desikan
cognitive	lateral frontal	pars opercularis	Desikan
cognitive	.	pars orbitalis	Desikan
cognitive	.	pars triangularis	Desikan
cognitive	Parietal	inferior parietal	Desikan
cognitive	.	superior parietal	Desikan
cognitive	.	precuneus	Desikan
cognitive	.	supramarginal	Desikan
cognitive	lateral temporal	superior temporal	Desikan
cognitive	.	middle temporal	Desikan
cognitive	medial temporal	hippocampal	Fischl
cognitive	.	entorhinal	Desikan
cognitive	.	parahippocampal	Desikan

Fischl refers to Fischl B, Salat DH, Busa E, et al. Whole brain segmentation: automated labeling of neuroanatomical structures in the human brain. Neuron 2002;33:341–355. Desikan refers to Desikan RS, Segonne F, Fischl B, et al. An automated labeling system for subdividing the human cerebral cortex on MRI scans into gyral based regions of interest. *NeuroImage* 2006;31:968–980.

### Statistical Analysis

Because of the potential laterality of emotional processing, we compared changes in volume across hemispheres in both the cross-sectional and longitudinal studies. No significant difference was found, further supporting our decision to perform the following studies bilaterally. Age, gender, and education were considered as covariates. In addition, because vascular risk factors can impact rates of decline and may impact regions differently [37–39], we also assessed the presence of diabetes, hypertension and hyperlipidemia as potential covariates, as well as body mass index (BMI) and extent of white matter hyperintensities on imaging, with a threshold of model inclusion if p < 0.15.

### Cross-Sectional Study

We used Stata v13.0 for all statistical analyses [40]. Significance was set at p < 0.05. Data are available in S1 File. Because of the significant volume difference in collective regions of emotion versus those of cognition, all volumes were converted to Z scores. A nonlinear locally weighted regression function was also performed and the results visually compared to confirm that a linear regression was a reasonable model for the cross-sectional and longitudinal datasets. For the cross-sectional analysis, linear regression was performed separately on the summed emotional brain volumes and the summed cognitive brain volumes as a function of age, with total intracranial volume (ICV) and gender as covariates. An age*subregion interaction term was introduced in order to statistically test for the difference between regional group coefficients. In addition to the combined analysis, volumes of contributing regions (the addends) were subsequently regressed against age as a secondary analysis. Again, an age*subregion interaction term was used to compare the regression coefficient of each subregion against the others, with a Bonferroni-adjusted significance threshold of p ≤ 0.001.

### Longitudinal Study

At least 6 months between scans was required. Otherwise, time between scans was assumed to be mixed and completely at random. To guard against the possibility of selective attrition, time spent between scans was regressed against regional volume at time 1, with a plan to include this time 1 volume as a covariate if significant relationships were found.

Mixed effects models were used to compare regional changes within individuals over time. These allowed random intercept and age slope, with cortical volume as the outcome. The model included main effects of time (time between the first and subsequent scan, in years) and region, with a time by region interaction. Gender, age and intracranial volume were included as covariates. The time by region interaction was used to investigate whether there was a significant difference in coefficients between emotional and cognitive groups.

As with the cross-sectional analysis, secondary analyses investigated changes in contributing subregions and between hemispheres. These consisted of mixed effects models for each subregion. The total volumes of the left and right hemisphere were compared in the same fashion. All longitudinal subregional analyses were considered exploratory and independent questions, and multiple comparisons were not performed.

## Results

Subject characteristics for cross-sectional and longitudinal analyses are outlined in Table 2.

**Table 2:** Baseline Demographic Data of Healthy Participants by Analysis Type.

	Cross-Sectional (n = 258)			Longitudinal (n = 84)		
	Mean	Std. Dev.	Range	Mean	Std. Dev.	Range
Age (in years)	72.0	6.8	55.1–99.6	70.1	6.4	55.1–87.2
Education (in years)	17.5 **	2.1	12–22	17.6	2.2	12–22
MMSE score	29.4	0.7	28–30	29.4 ^††^	4.71	28–30
Women (%)	55.8			61.9		
Handedness (%)	85.6			88.1		
Race (% Caucasian)	95.0*			97.6 ^†^		

Demographic characteristics of healthy research participants. Those participants from the cross-sectional study who received more than one scan were included in both the cross-sectional and longitudinal studies.

*238 responders.

**176 responders.

^†^ 83 responders.

^††^ 62 responders.

### Cross-sectional Analysis

**Fig 2:**
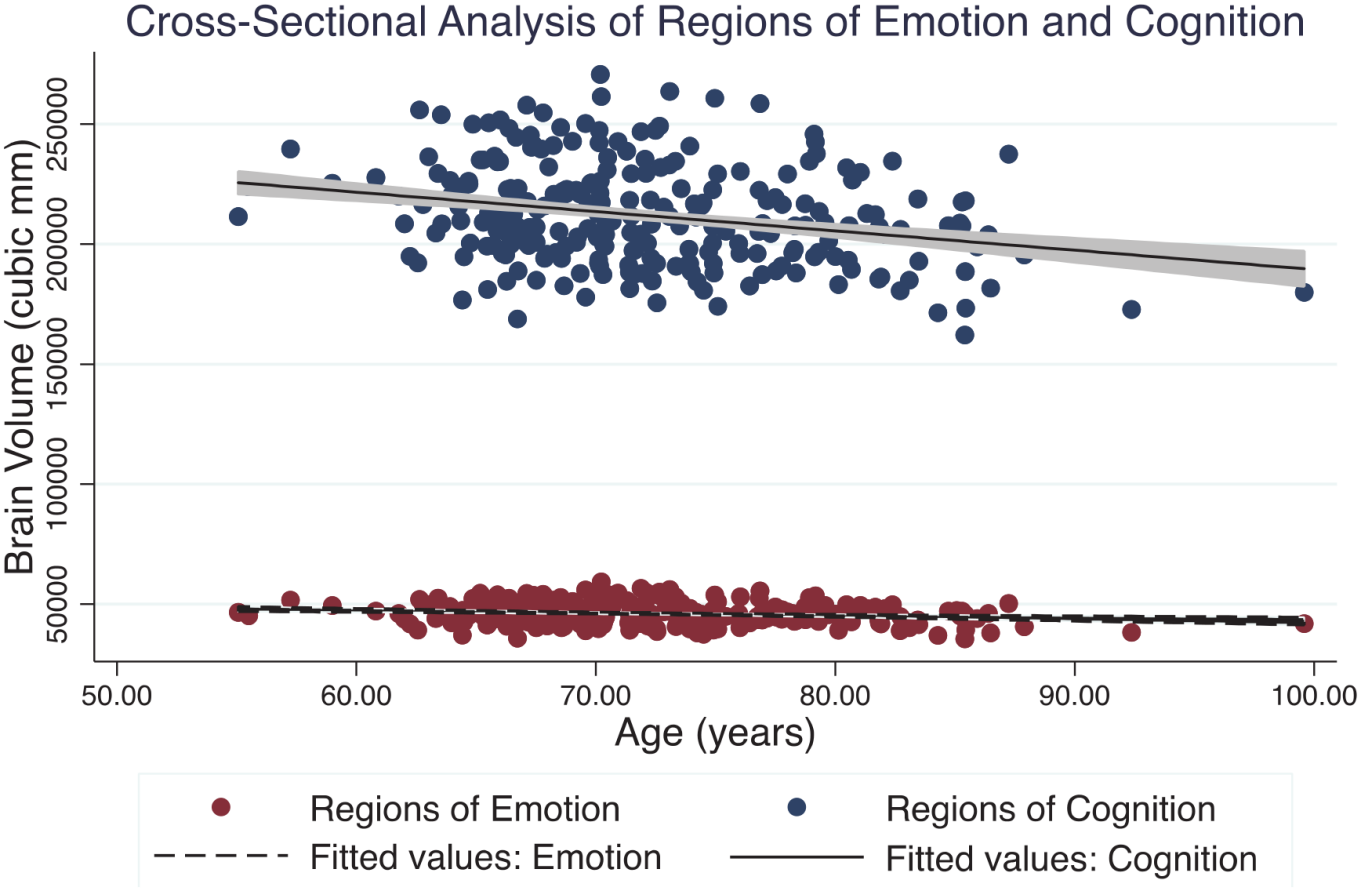
Cross-Sectional Analysis of Regions of Emotion and Cognition. Absolute brain volume in collective regions associated with cognition compared to those associated with emotion as a function of healthy aging research participants’ age in years. There was an estimated 0.51% per year absolute volume loss in regions of emotion and 0.60% per year absolute volume loss in regions of cognition. Note that while absolute volumes are shown here for clear visualization of the two groups, for statistical comparison of the association between age and volume between regions of emotion and cognition, volumes were converted to Z-scores in order to normalize the baseline difference in absolute volume between groups. No significant proportional difference was found between the cognitive or emotional regions in volume loss as a function of age.

Age was associated with decreased brain volume both in regions of cognition ($\hat{\beta }$ **= -**0.05, R^2^ = 0.61, p < 0.001, CI [-0.06, -0.04]) and in regions of emotion ($\hat{\beta }$ **= -**0.04, R^2^ = 0.64, p < 0.001, CI [-0.05, -0.03]) (Fig 2).

Cross-sectional analysis showed no significant interaction between age and region type ($\hat{\beta }$ **=** 0.01, R^2^ = 0.62, p = 0.096, CI [-.002, 0.03]) after adjusting for intracranial volume and gender. While a p value < 0.1 might be considered a trend, any effect size for the interaction is very small (eta^2^ = 0.005). Education, vascular risk factors and white matter hyperintensities did not contribute significantly to the model (all p > 0.20).

On subgroup analysis, the rostral ACC had the smallest association between volume and age, with the most significant interaction coefficient found in the medial temporal lobe (Table 3). Comparisons between groups in the interaction between age and volume suggest the ACC has a significantly smaller association between age and volume when compared with the amygdala ($\hat{\beta }$ **=** 0.04, p<0.001, CI[0.02, 0.06]), nucleus accumbens ($\hat{\beta }$ **=** 0.06, p<0.001, CI [-0.08, -0.04]), parietal lobes ($\hat{\beta }$ **=** 0.03, p = 0.001, CI {-0.05, 0.01]), medial temporal lobes ($\hat{\beta }$ **=** = -0.04, p<0.001, CI[-0.07, -0.03]), and lateral temporal lobes ($\hat{\beta }$ **=** = -0.04, p<0.001, CI[-0.06, -0.02), after adjusting for multiple comparisons. In contrast to the relative preservation of the ACC, the nucleus accumbens had a stronger relationship between age and decreasing volume when compared to the insula ($\hat{\beta }$ **=** = 0.05, p<0.001, CI [0.03, 0.07]), the orbitofrontal cortex ($\hat{\beta }$ **=** = 0.04, p<0.001, CI[0.02, 0.06]), the middle frontal cortex ($\hat{\beta }$ **=** = 0.04, p<0.001, CI[0.02, 0.06]), as well as the ACC. The medial temporal lobe differed significantly from the insula ($\hat{\beta }$ **=** = -0.04, p<0.001, CI[-0.06, -0.02]) and middle frontal cortex ($\hat{\beta }$ **=** = -0.04, p<0.001, CI[-0.06, -0.02]).

**Table 3:** Regression Coefficients for Sub-Regions by Analysis Type.

	Cross-Sectional (by age)			Longitudinal (by time)		
	β	Std. Err.	95% Conf. Interval	β	Std. Err.	95% Conf. Interval
Amygdala	-0.05	0.01	-0.07–-0.03	0.23	0.03	0.16–0.29
Rostral ACC	-0.02	0.01	-0.04–-0.001	-0.10	0.02	-0.13–-0.06
Insula	-0.03	0.01	-0.04–-0.01	-0.14	0.02	-0.19–-0.09
Nucleus Accumbens	-0.06	0.01	-0.08–-0.04	0.11	0.01	0.02–0.19
OFC	-0.04	0.01	-0.05–-0.02	-0.09	0.02	-0.13–-0.05
Lateral Frontal	-0.04	0.01	-0.06–-0.02	-0.10	0.02	-0.14–-0.06
Middle Frontal	-0.03	0.01	-0.05–-0.02	-0.10	0.02	-0.13–-0.07
Parietal	-0.05	0.01	-0.07–-0.03	-0.13	0.02	-0.17–-0.10
Lateral Temporal	-0.05	0.01	-0.07–-0.03	-0.08	0.01	-0.10–-0.05
Medial Temporal	-0.07	0.01	-0.09–-0.05	0.01	0.02	-0.02–0.04

Subgroup analysis of cross-sectional and longitudinal studies for regions of emotion (top), and regions of cognition (bottom). Units are in millimeters cubed per year of age for cross-sectional data, or by year since the original MRI scan for longitudinal data. The cross-sectional analysis is adjusted for ICV and gender. The longitudinal analysis adjusted for ICV, baseline regional volume, gender and age.

### Longitudinal Analysis

Preliminary data inspection showed that a longer period of time between scans was positively correlated with volume in the amygdala ($\hat{\beta }$ = 1.12, p = 0.002, CI [0.42, 1.82]) as well as the nucleus accumbens ($\hat{\beta }$ = 1.35, p < 0.001, CI [0.65, 2.1]) at time 1. Baseline volume was therefore included as a covariate in further longitudinal analyses. Again, education, vascular risk factors and white matter hyperintensities did not contribute significantly to the model (all p > 0.20). Mixed effects modeling confirmed the non-independence of longitudinal volumetric data between individuals ($\hat{\beta }$ = 0.95, p < 0.001, CI [0.8, 1.1]).

**Fig 3:**
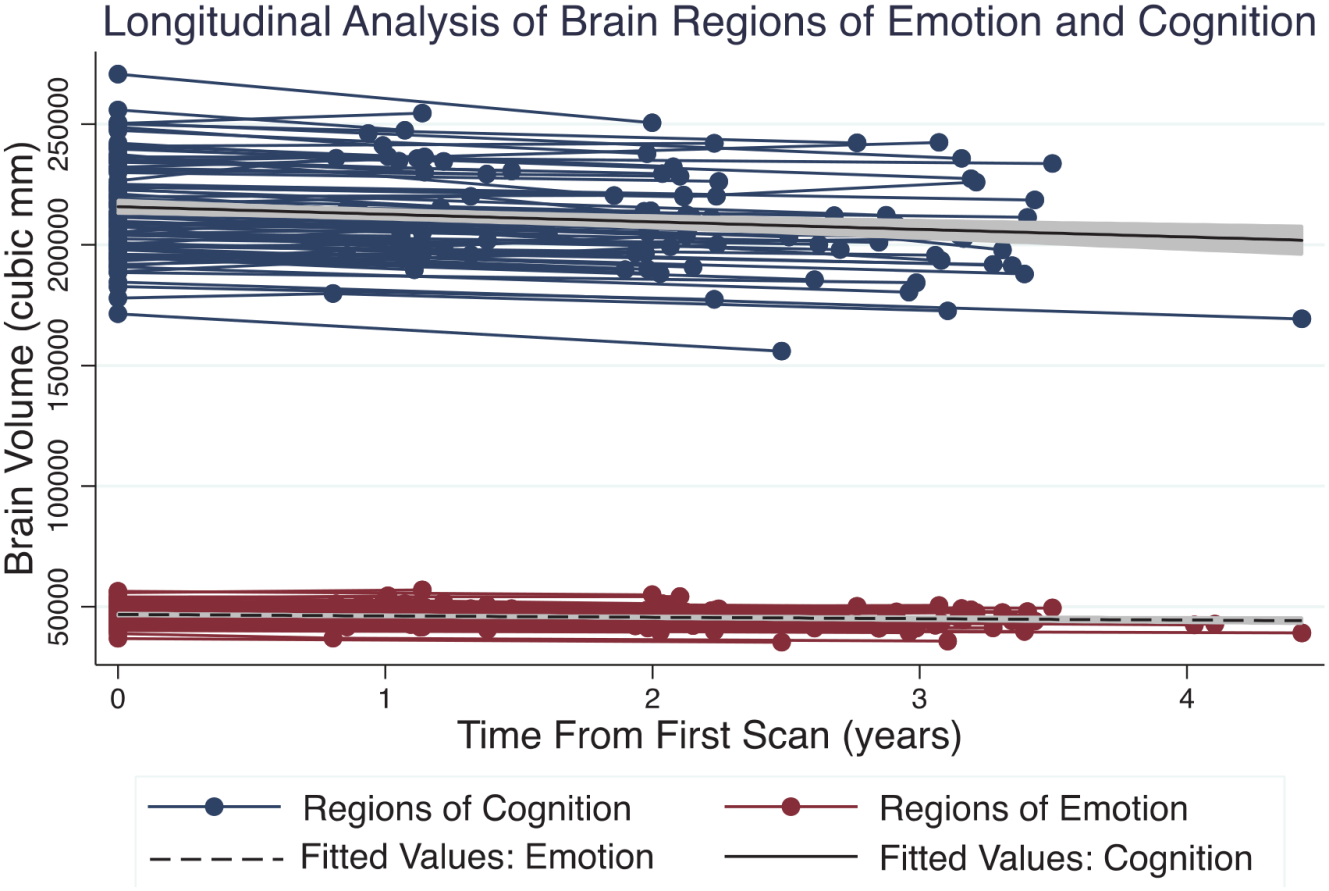
Longitudinal Analysis of Brain Regions of Emotion and Cognition. Absolute volume of collective cognitive and emotional regions in healthy aging participants who were scanned over time. While volumes were converted to Z-scores for rate comparisons analysis in order to adjust for baseline differences in absolute volume between regions of cognition and emotion, absolute brain volumes are here depicted for visualization of individual slopes. In absolute volume, regions of cognition lost approximately 0.65% per year, and regions of emotion lost approximately 0.46% per year. No statistically significant proportional difference was found between brain regions of cognition and emotion in rates of volume loss over time.

Significant volume loss was found over time in regions of emotion ($\hat{\beta }$ = -0.05, p = 0.003, CI [-0.08, -0.03]) as well as cognition ($\hat{\beta }$ = -0.07, p < 0.001, CI [-0.09, -0.02]) (Fig 3).

When comparing regression slopes, no significant difference in the time/volume interaction term was found between cognitive and emotional collectives ($\hat{\beta }$ = 0.01, p = 0.70, CI [-0.04, 0.06]). As with the cross-sectional studies, sub-regions were investigated as well to assess their potential contribution to the observed trend. In this analysis, two regions of emotion, the nucleus accumbens and the amygdala, demonstrated statistically significant positive volume changes with time. Including baseline volumes as a covariate did not alter this trend. Among regions of cognition, the medial temporal lobe did not show significant age-related volume loss.

## Discussion

The purpose of this study was to examine whether differing rates of volume loss between brain regions commonly associated with emotion as opposed to cognition might contribute to preserved emotional health with aging. We used two different study designs (cross-sectional and longitudinal) to approach the question of how cognitive and emotional brain regions change with time. Our primary analyses found no difference between emotional and cognitive brain volumes as a function of age or time’s passage.

One of the strengths of this study was our use of both cross-sectional and longitudinal approaches. The cross-sectional design included a larger sample size. The longitudinal approach examines fewer individuals, but has the benefit of allowing better causal inference between volume and time, rather than relying on age as a proxy for the passage of time.

Subgroup analyses within both the longitudinal and cross-sectional studies support previous reports of relative preservation of the rostral anterior cingulate cortex, with smaller rates of decline compared to many other cortical regions (Table 3). Relative preservation of the ACC has been previously connected with the emotion preservation of aging [6], and with the positivity effect in particular [17]. The term “positivity effect” describes the tendency of elders to preferentially attend to positive over negative stimuli in a variety of contexts, including facial expressions, word lists [41], and emotional images [42–44]. Elders tend to recall more positive than negative stimuli in working, short-term, and autobiographical memories [43, 45, 46]. The use of such selective attention to avoid of negative stimuli another common emotional regulatory strategy used by elderly adults, which may relate to this positivity effect [6, 15, 17, 47]. Brassen and colleagues have correlated the anterior cingulate with such selective attention using functional connectivity. While the region identified by Brassen and colleagues is at the dorsal-most part of the ACC included in our partitioning, in an area many would associate with cognitive processes, the entire cingulate, both ventral and dorsal, has been shown to be relatively preserved in previous imaging studies [14, 15]. Furthermore, a more ventral portion has been associated with the positivity effect by Ritchey et al during a task demanding concentrated focus, akin to positive reappraisal [48].

The cross-sectional analysis also showed a significantly greater negative association between age and volume in the nucleus accumbens compared to other brain regions such as the rostral ACC, insula, orbitofrontal cortex, and the middle frontal lobe. Previous studies have also demonstrated age-related volume loss in the nucleus accumbens [49]. Unlike the ACC, little has been written on the accumbens and preservation of emotional functioning in aging, with an emphasis instead placed on decision-making [50, 51]. While tempted to speculate on how loss of this structure might place a greater processing burden on cortex, thereby driving a preference for positive emotion [18], the nucleus accumbens is a complex structure, and consequences of its loss are also likely to be complex. For example, while the nucleus accumbens has been traditionally tied with reward systems, it also mediates aversive reactions [52]. Further research will be needed to correlate degeneration of the nucleus accumbens with cognitive and emotional changes in healthy aging.

Volume in brain regions of cognition and emotion declined as a function of age in the cross-sectional study, and as a function of time in the longitudinal study, to a degree similar to overall cortical volume loss demonstrated in prior studies [53]. While the collective trajectories of cognitive versus emotional brain volumes are consistent between our cross-sectional and longitudinal studies, some sub-regions appear discordant on sub-regional analysis. Chiefly, the medial temporal lobe, amygdala and nucleus accumbens appear to gain volume over time. Previous studies have shown conflicting evidence of volume changes in these regions over time [14, 54]. On the other hand, Freesurfer has been previously noted to have questionable scan-rescan reliability in the parcellation of small, subcortical structures [55]. The apparent sub-regional volume gain may reflect a greater amount of variability in volumetric analysis of relatively small or medial structures using our techniques, which may be especially important with the smaller sample size found in the longitudinal group. In the comparison between cognitive and emotional regions collectively, any consequent bias present in the emotion regions would likely contribute to apparent emotional preservation. Failure to reject the null hypothesis in spite of such potential bias bolsters the negative finding between cognitive and emotional volume changes collectively. We consider the cross-sectional data to be more reliable within our study, and therefore limited between-subgroup analysis to the cross-sectional portion.

Other potential weaknesses to this study design include the subjective nature of regions selected as “emotional” or “cognitive.” As we know of no widely accepted battery of emotional functioning, this study instead relies on established correlations found in scientific literature. Because cognitive and emotional changes with age, as well as their neuroanatomical underpinnings, are complex and interwoven [56], selection of regions that could be congregated reliably into either emotional or cognitive group was a particular challenge of this research. While we recognize the imperfection of our regional groupings, absolute parcellation of cognitive and emotional functions is impossible, necessitating reliance on best judgment. Another potential limitation is the possibility that some of our healthy population in fact has histopathological changes associated with neurodegenerative disease. Our definition of this population as healthy agers must be recognized as strictly clinical.

In addition to our collective approach and the use of two different study designs, strengths of this research include a large sample size and relative consistency of magnetic resonance imaging and analysis techniques within a healthy aging population. Future studies could examine differential rates of volume loss within neuroanatomical regions over more visits to allow for imputation, as well as correlate active measures of emotion and cognition to confirm corresponding neuropsychological performance. Functional connectivity or other techniques could also be explored.

## Conclusions

In summary, our results suggest that during healthy aging, brain regions commonly associated with emotional processing collectively undergo a rate of decline proportional to those of cognitive regions. Clustering all regions of emotion versus those of cognition does not provide sufficient specificity to provide a biological explanation for emotional preservation with aging. More detailed cross-sectional comparisons between subregions suggests that some regions of emotion such as the anterior cingulate cortex may be truly spared, whereas others such as the nucleus accumbens may be particularly susceptible to age-dependent volume loss. Further studies may further explore how these patterns of volume loss contribute to emotional functioning by correlation with dedicated cognitive and emotional tests.

## Supporting Information

S1 File Dataset. A de-identified dataset used in the analyses presented.
(XLSX)

## References

[1] Reuter-Lorenz PA, Lustig C. Brain aging: reorganizing discoveries about the aging mind. Current opinion in neurobiology. 2005;15(2):245–51. 15831410 10.1016/j.conb.2005.03.016

[2] Stemmler M, Petermann F, Daseking M, Siebert J, Schott H, Lehfeld H, et al (The assessment and course of development of cognitive abilities in the elderly). Gesundheitswesen. 2013;75(11):761–7. 10.1055/s-0033-1357164 24163218

[3] Buckner RL. Memory and Executive Function in Aging and AD. Neuron. 2004;44(1):195–208. 15450170 10.1016/j.neuron.2004.09.006

[4] Bennett DA, Wilson RS, Boyle PA, Buchman AS, Schneider JA. Relation of neuropathology to cognition in persons without cognitive impairment. Annals of neurology. 2012;72(4):599–609. 10.1002/ana.23654 23109154 PMC3490232

[5] Charles ST. Strength and vulnerability integration: a model of emotional well-being across adulthood. Psychological bulletin. 2010;136(6):1068–91. 10.1037/a0021232 21038939 PMC3059514

[6] Mather M. The emotion paradox in the aging brain. Annals of the New York Academy of Sciences. 2012;1251:33–49. 10.1111/j.1749-6632.2012.06471.x 22409159 PMC3395773

[7] Charles ST, Carstensen LL. Social and emotional aging. Annual review of psychology. 2010;61:383–409. 10.1146/annurev.psych.093008.100448 19575618 PMC3950961

[8] Jeste DV, Oswald AJ. Individual and Societal Wisdom: Explaining the Paradox of Human Aging and High Well-Being. Psychiatry. 2014. 10.1521/psyc.2014.77.4.317 25386770

[9] Jopp D, Rott C. Adaptation in very old age: exploring the role of resources, beliefs, and attitudes for centenarians' happiness. Psychology and aging. 2006;21(2):266–80. 16768574 10.1037/0882-7974.21.2.266

[10] Sze JA, Gyurak A, Goodkind MS, Levenson RW. Greater emotional empathy and prosocial behavior in late life. Emotion. 2012;12(5):1129–40. 10.1037/a0025011 21859198 PMC3766763

[11] Levenson RW. Expressive, physiological and subjective changes in emotion In: Qualls SH, Abeles N, editors. Psychology and the aging revolution: How we adapt to longer life. Washington, DC: American Psychological Association; 2000 p. 123–40.

[12] Seider BH, Shiota MN, Whalen P, Levenson RW. Greater sadness reactivity in late life. Social cognitive and affective neuroscience. 2011;6(2):186–94. 10.1093/scan/nsq069 20650943 PMC3073392

[13] Shiota MN, Levenson RW. Effects of aging on experimentally instructed detached reappraisal, positive reappraisal, and emotional behavior suppression. Psychology and aging. 2009;24(4):890–900. 10.1037/a0017896 20025404 PMC2805117

[14] Fjell AM, McEvoy L, Holland D, Dale AM, Walhovd KB. Brain changes in older adults at very low risk for Alzheimer's disease. The Journal of neuroscience: the official journal of the Society for Neuroscience. 2013;33(19):8237–42. 23658162 10.1523/JNEUROSCI.5506-12.2013 PMC4050197

[15] Fjell AM, Westlye LT, Amlien I, Espeseth T, Reinvang I, Raz N, et al High consistency of regional cortical thinning in aging across multiple samples. Cereb Cortex. 2009;19(9):2001–12. 10.1093/cercor/bhn232 19150922 PMC2733683

[16] Mann SL, Hazlett EA, Byne W, Hof PR, Buchsbaum MS, Cohen BH, et al Anterior and posterior cingulate cortex volume in healthy adults: effects of aging and gender differences. Brain research. 2011;1401:18–29. 10.1016/j.brainres.2011.05.050 21669408 PMC3134959

[17] Brassen S, Gamer M, Buchel C. Anterior cingulate activation is related to a positivity bias and emotional stability in successful aging. Biological psychiatry. 2011;70(2):131–7. 10.1016/j.biopsych.2010.10.013 21183158

[18] Labouvie-Vief G, hn D, Studer J. Dynamic integration of emotion and cognition: equilibrium regulation in development and aging In: Lerner R, Lamb M, Freund A, editors. The Handbook of Life-Span Development. 2 Hoboken: John Wiley & Sons, Inc.; 2010 p. 79–115.

[19] Johnsrude IS, Owen AM, White NM, Zhao WV, Bohbot V. Impaired preference conditioning after anterior temporal lobe resection in humans. The Journal of neuroscience: the official journal of the Society for Neuroscience. 2000;20(7):2649–56. 10729345 10.1523/JNEUROSCI.20-07-02649.2000 PMC6772234

[20] Schoenbaum G, Setlow B. Lesions of nucleus accumbens disrupt learning about aversive outcomes. The Journal of neuroscience: the official journal of the Society for Neuroscience. 2003;23(30):9833–41. 14586012 10.1523/JNEUROSCI.23-30-09833.2003 PMC6740900

[21] Fischl B, Salat DH, Busa E, Albert M, Dieterich M, Haselgrove C, et al Whole brain segmentation: automated labeling of neuroanatomical structures in the human brain. Neuron. 2002;33(3):341–55. 11832223 10.1016/s0896-6273(02)00569-x

[22] Desikan RS, Segonne F, Fischl B, Quinn BT, Dickerson BC, Blacker D, et al An automated labeling system for subdividing the human cerebral cortex on MRI scans into gyral based regions of interest. NeuroImage. 2006;31(3):968–80. 16530430 10.1016/j.neuroimage.2006.01.021

[23] Straube T, Miltner WH. Attention to aversive emotion and specific activation of the right insula and right somatosensory cortex. NeuroImage. 2011;54(3):2534–8. 10.1016/j.neuroimage.2010.10.010 20946962

[24] Duerden EG, Arsalidou M, Lee M, Taylor MJ. Lateralization of affective processing in the insula. NeuroImage. 2013;78:159–75. 10.1016/j.neuroimage.2013.04.014 23587690

[25] Goldman-Rakic P. The prefrontal landscape: implications of functional architecture for understanding human mentation and the central executive. Philosophical Transactions of the Royal Society of London. 1996;351:1445–53. 8941956 10.1098/rstb.1996.0129

[26] Cabeza R, Ciaramelli E, Moscovitch M. Cognitive contributions of the ventral parietal cortex: an integrative theoretical account. Trends in cognitive sciences. 2012;16(6):338–52. 10.1016/j.tics.2012.04.008 22609315 PMC3367024

[27] Jefferies E. The neural basis of semantic cognition: converging evidence from neuropsychology, neuroimaging and TMS. Cortex; a journal devoted to the study of the nervous system and behavior. 2013;49(3):611–25. 10.1016/j.cortex.2012.10.008 23260615

[28] Duncan J. The structure of cognition: attentional episodes in mind and brain. Neuron. 2013;80(1):35–50. 10.1016/j.neuron.2013.09.015 24094101 PMC3791406

[29] Gross RG, Grossman M. Executive resources. Continuum (Minneap Minn). 2010;16(4 Behavioral Neurology):140–52. 22810519 10.1212/01.CON.0000368266.46038.0e PMC3652226

[30] Quirk GJ, Armony JL, LeDoux JE. Fear conditioning enhances different temporal components of tone-evoked spike trains in auditory cortex and lateral amygdala. Neuron. 1997;19(3):613–24. 9331352 10.1016/s0896-6273(00)80375-x

[31] Wilensky AE, Schafe GE, LeDoux JE. Functional inactivation of the amygdala before but not after auditory fear conditioning prevents memory formation. The Journal of neuroscience: the official journal of the Society for Neuroscience. 1999;19(24):RC48. 10594092 10.1523/JNEUROSCI.19-24-j0006.1999 PMC6784952

[32] Goodkind MS, Sollberger M, Gyurak A, Rosen HJ, Rankin KP, Miller B, et al Tracking emotional valence: the role of the orbitofrontal cortex. Human brain mapping. 2012;33(4):753–62. 10.1002/hbm.21251 21425397 PMC3217132

[33] Sturm VE, Sollberger M, Seeley WW, Rankin KP, Ascher EA, Rosen HJ, et al Role of right pregenual anterior cingulate cortex in self-conscious emotional reactivity. Social cognitive and affective neuroscience. 2013;8(4):468–74. 10.1093/scan/nss023 22345371 PMC3624960

[34] Cardinal RN, Parkinson JA, Hall J, Everitt BJ. Emotion and motivation: the role of the amygdala, ventral striatum, and prefrontal cortex. Neuroscience and biobehavioral reviews. 2002;26(3):321–52. 12034134 10.1016/s0149-7634(02)00007-6

[35] Medford N, Critchley HD. Conjoint activity of anterior insular and anterior cingulate cortex: awareness and response. Brain structure & function. 2010;214(5–6):535–49. 20512367 10.1007/s00429-010-0265-x PMC2886906

[36] Binney RJ, Embleton KV, Jefferies E, Parker GJM, Ralph MAL. The ventral and inferolateral aspects of the anterior temporal lobe are crucial in semantic memory: evidence from a novel direct comparison of distortion-corrected fMRI, rTMS, and semantic dementia. Cerebral Cortex. 2010;20(11):2728–38. 10.1093/cercor/bhq019 20190005

[37] Moran C, Phan TG, Chen J, Blizzard L, Beare R, Venn A, et al Brain atrophy in type 2 diabetes: regional distribution and influence on cognition. Diabetes care. 2013;36(12):4036–42. 10.2337/dc13-0143 23939539 PMC3836136

[38] Bilello M, Doshi J, Nabavizadeh SA, Toledo JB, Erus G, Xie SX, et al Correlating Cognitive Decline with White Matter Lesion and Brain Atrophy Magnetic Resonance Imaging Measurements in Alzheimer's Disease. Journal of Alzheimer's disease: JAD. 2015;48(4):987–94. 10.3233/JAD-150400 26402108 PMC4637168

[39] Dong C, Nabizadeh N, Caunca M, Cheung YK, Rundek T, Elkind MS, et al Cognitive correlates of white matter lesion load and brain atrophy: the Northern Manhattan Study. Neurology. 2015;85(5):441–9. 10.1212/WNL.0000000000001716 26156514 PMC4534076

[40] StataCorp. Stata Statistical Software: Release 13. College Station, TX: StataCorp LP; 2013.

[41] Piguet O, Connally E, Krendl AC, Huot JR, Corkin S. False memory in aging: effects of emotional valence on word recognition accuracy. Psychology and aging. 2008;23(2):307–14. 10.1037/0882-7974.23.2.307 18573005

[42] Mather M, Carstensen LL. Aging and attentional biases for emotional faces. Psychological science. 2003;14(5):409–15. 12930469 10.1111/1467-9280.01455

[43] Charles ST, Mather M, Carstensen LL. Aging and emotional memory: the forgettable nature of negative images for older adults. Journal of experimental psychology General. 2003;132(2):310–24. 12825643 10.1037/0096-3445.132.2.310

[44] Isaacowitz DM, Wadlinger HA, Goren D, Wilson HR. Selective preference in visual fixation away from negative images in old age? An eye-tracking study. Psychology and aging. 2006;21(1):40–8. 16594790 10.1037/0882-7974.21.1.40

[45] Kennedy Q, Mather M, Carstensen LL. The role of motivation in the age-related positivity effect in autobiographical memory. Psychological science. 2004;15(3):208–14. 15016294 10.1111/j.0956-7976.2004.01503011.x

[46] Schlagman S, Schulz J, Kvavilashvili L. A content analysis of involuntary autobiographical memories: examining the positivity effect in old age. Memory. 2006;14(2):161–75. 16484107 10.1080/09658210544000024

[47] Gyurak A, Gross JJ, Etkin A. Explicit and implicit emotion regulation: a dual-process framework. Cognition & emotion. 2011;25(3):400–12. 21432682 10.1080/02699931.2010.544160 PMC3280343

[48] Ritchey M, Bessette-Symons B, Hayes SM, Cabeza R. Emotion processing in the aging brain is modulated by semantic elaboration. Neuropsychologia. 2011;49(4):640–50. 10.1016/j.neuropsychologia.2010.09.009 20869375 PMC3042541

[49] Long X, Liao W, Jiang C, Liang D, Qiu B, Zhang L. Healthy aging: an automatic analysis of global and regional morphological alterations of human brain. Academic radiology. 2012;19(7):785–93. 10.1016/j.acra.2012.03.006 22503890

[50] Samanez-Larkin GR, Kuhnen CM, Yoo DJ, Knutson B. Variability in nucleus accumbens activity mediates age-related suboptimal financial risk taking. The Journal of neuroscience: the official journal of the Society for Neuroscience. 2010;30(4):1426–34. 20107069 10.1523/JNEUROSCI.4902-09.2010 PMC2821055

[51] Meeks TW, Jeste DV. Neurobiology of wisdom: a literature overview. Archives of general psychiatry. 2009;66(4):355–65. 10.1001/archgenpsychiatry.2009.8 19349305 PMC3698847

[52] Berridge KC, Kringelbach ML. Neuroscience of affect: brain mechanisms of pleasure and displeasure. Current opinion in neurobiology. 2013;23(3):294–303. 10.1016/j.conb.2013.01.017 23375169 PMC3644539

[53] Hedman AM, van Haren NE, Schnack HG, Kahn RS, Hulshoff Pol HE. Human brain changes across the life span: a review of 56 longitudinal magnetic resonance imaging studies. Human brain mapping. 2012;33(8):1987–2002. 10.1002/hbm.21334 21915942 PMC6870052

[54] Cherbuin N, Sachdev PS, Anstey KJ. Mixed handedness is associated with greater age-related decline in volumes of the hippocampus and amygdala: the PATH through life study. Brain and behavior. 2011;1(2):125–34. 10.1002/brb3.24 22399092 PMC3236539

[55] Morey RA, Selgrade ES, Wagner HR 2nd, Huettel SA, Wang L, McCarthy G. Scan-rescan reliability of subcortical brain volumes derived from automated segmentation. Human brain mapping. 2010;31(11):1751–62. 10.1002/hbm.20973 20162602 PMC3782252

[56] John YJ, Bullock D, Zikopoulos B, Barbas H. Anatomy and computational modeling of networks underlying cognitive-emotional interaction. Frontiers in human neuroscience. 2013;7:101 10.3389/fnhum.2013.00101 23565082 PMC3613599

